# Addressing inequities in research for early to mid-career women scientists in low- and middle-income countries: “Supporting Women in Science” programme

**DOI:** 10.3389/fgwh.2024.1386809

**Published:** 2024-11-28

**Authors:** Jai K. Das, Masooma Raza, Zahra Ali Padhani, Narjis Fatima Hussain, Jose Villar, Stephen Kennedy, Zulfiqar A. Bhutta

**Affiliations:** ^1^ Institute for Global Health and Development, Aga Khan University, Karachi, Pakistan; ^2^ Oxford Maternal and Perinatal Health Institute, University of Oxford, Oxford, United Kingdom; ^3^ SickKids Centre for Global Child Health, University of Toronto, Toronto, ON, Canada

**Keywords:** capacity building, gender equity, distance learning, academic programme, mentorship

## Abstract

**Introduction:**

The gender disparities and inequities faced by women in academia are widespread, especially in low- and middle-income countries (LMICs). The scholarly output of women scientists remains significantly lower than that of men due to limited opportunities. This widening gap has significant implications for policy-making and prioritizing agendas. The Supporting Women in Science (SWIS) programme aims to address these barriers by enhancing research capacity among early- and mid-career women scientists from LMIC regions such as South-Central Asia and East Africa, in bespoke areas of health and health-related sustainable development goals (HHSDGs).

**Methods:**

The SWIS programme utilizes online and distance learning with a self-paced approach. Applications are accepted on a rolling basis, through a pre-defined eligibility criterion. Phase I involves online self-learning courses covering a core and elective curriculum over 6 months which is then evaluated in Phase II. Eligible candidates then move to Phase III, a mentored fellowship where they develop research proposals and receive funding for research project development, implementation, and evaluation. The rigorous reporting and monitoring mechanisms track compliance and progress. The online format, offered at no cost, enhances program accessibility, particularly in the post-COVID era. Additionally, SWIS prioritizes mentorship by selecting experienced professionals with strong research backgrounds and mentorship skills to guide participants. The programme evaluation will be based on selected success metrics including program completion ratio, funding opportunities availed by the participants, and generated scholarly output and presentations at key events.

**Discussion and conclusion:**

Securing grant funding is pivotal for career advancement, yet women applicants face greater challenges as compared to men. The SWIS programme not only equips participants with knowledge and skills but also facilitates practical application through a simulated process, enabling participants to pursue future funding opportunities. Capacity-building initiatives like SWIS are crucial interventions to empower women scientists, foster equitable representation in academia, and create inclusive research environments and the programme acts as a steppingstone for future global leaders.

## 1 Introduction

The existing disparities and gender inequities for women in academia are a universal phenomenon, but the gaps are particularly profound in low- and middle- income countries (LMICs) (1–3). The UN Educational, Scientific and Cultural Organization (UNESCO)'s report reveals that women comprise less than 30% of the world's researchers, with the lowest proportions in South and West Asia (19%) and East Asia and the Pacific (24%) (4). These disparities directly relate to the United Nations Sustainable Development Goals (SDGs), particularly the SDG 4: Quality Education and SDG 5: Gender Equality. As such, SDG 4 aims to ensure inclusive and equitable quality education and promote lifelong learning opportunities for all while SDG 5 aims to achieve gender equality and empower women and girls, emphasizing the importance of equal opportunities in all spheres, including education and employment.

However, despite recent increases in women researcher numbers, their scholarly output (number of publications and international collaborations) remains much lower than that of their male counterparts, reflecting limited opportunities for training, employment, and academic career enhancement (5). In high-quality journals listed in the Nature Index covering the categories Life Science, Multidisciplinary, Earth & Environmental and Chemistry; only 29.8% of all authors, and 33.1% of first, 31.8% of co- and 18.1% of senior authors were women (6). More specifically, a significantly large negative correlation was observed between the 5-Year-Impact-Factor of a journal and female authorship (6). Another study using PubMed and Web of Science concluded that out of 7,370 publications from 2008 to 2018, across 11 specialty and general journals for critical care medicine, only 30.4% had female first authors and 15.5% female senior authors (7). Hence, prominent authorship positions in high-impact publications including in the area of life and health sciences and STEM are still being held predominantly by men.

In academia, publications and secured funding are two metrics of success. With the well-established importance of securing grants, it is worthwhile to note that female applicants are less likely to receive research funding than men (8). A retrospective analysis of grant submissions to the Canadian Institutes of Health Research showed women had significantly lower grant success compared with men, with substantial heterogeneity (8). Hence, the existing bias in grant review processes and the structural disparities put female scientists at a huge disadvantage.

A further milestone in academic career progression is achieving tenure, which provides professors with permanent employment, and is inherently linked to academic freedom (9), associated prestige, stability, and other benefits (10). A study exploring gender disparities in tenure status concluded that in 2019, nearly 60% of full-time university faculty members in Canada were men and that female faculty members were less likely to hold tenured positions, with only 63% of women in such roles compared to 75% of men (9).

Further, perceptions of fairness in hiring and promotions varies significantly by gender, as about 20% of female faculty disagreed or strongly disagreed that hiring practices were fair and equitable at their institutions, compared to only 12% for male faculty (9). Similarly, women (23%) were more likely than men (14%) to express dissatisfaction with the fairness of promotion processes (9).

Consequently, key positions in research and academia are usually occupied by men while women take less prestigious teaching positions (1, 9, 10). Entrenched patriarchal attitudes, the perception of women's major role in child-rearing, the dearth of re-entry friendly policies after maternity-related breaks, and the lack of gender-sensitive policies in the workplace all contribute as well to the enhanced gender gap (1). Studies have shown that even when qualifications are identical, male candidates are more frequently selected for interviews and job offers (11, 12) and when offered, women have lower start-up packages and salaries compared to their male counterparts (13).

In addition to the professional challenges, family responsibilities often pose a greater burden for women in academia than for men. Women with children or other dependents can face additional obstacles during the hiring process, while men in similar situations are less affected (14, 15). As a result, women may prioritize positions offering better access to health care, family leave, and childcare. Furthermore, women are generally more willing to make career sacrifices for family, such as declining or leaving tenure-track positions in favour of more flexible roles, leading to their overrepresentation in non-tenure-track or part-time positions (16).

These gender gaps are even more prominent in LMICs due to interlaced social and cultural norms (1). For example, a study in India found that men dominated research publications in 26 broad areas of scholarship including STEM and broader fields of medicine, nursing, social sciences, and health (3). However, the U.S. data in the same disciplines were markedly different, suggesting that gender imbalances stem more from cultural attitudes and gender-sensitive policies than innate differences or gender-based preferences (3). This persistent and, at times, widening gender gap in health-related research, has broad implications on gender-sensitive policy-making and prioritising agendas that address the gender dimensions of health, including issues that disproportionately affect women (1).

We seek to address some of these issues of gender disparity in research and scholarly outputs through an innovative programme of research capacity development that builds on the extensive research networks established by the INTERGROWTH-21st (17), INTERBIO-21st (18) and INTERCOVID (19) consortia. These programs, developed by the Oxford Maternal and Perinatal Health Institute, represent interconnected initiatives aimed at enhancing maternal and child health outcomes worldwide. They share a collective commitment to advancing evidence-based strategies for improving perinatal care, aligning closely with the research focus areas in LMICs. Through their extensive resources and networks, these programs provide foundational knowledge and methodological frameworks that can be integrated into program curriculum. The collaboration with researchers from these initiatives further strengthens the network, offering mentorship and expertise that are invaluable for fostering women's contributions to maternal and child health research and development globally. Overall, the aim is to enhance existing research capacity in the field of women's reproductive and perinatal health and expand to other areas of health and health-related Sustainable Development Goals (HHSDGs) (20) by providing additional training to early- to mid-career female researchers.

The “Supporting Women in Science” (SWIS) programme, led by the Institute for Global Health and Development at the Aga Khan University, Pakistan; Center of Global Child Health at SickKids, Canada, and University of Oxford, UK, offers early- and mid-career female researchers in South-Central Asia and East Africa the opportunity to progress their careers and develop professionally as future leaders across these key LMIC regions. The programme, which will potentially reduce current inequities and promote gender equality in science, capitalises on the past experiences of the Wellcome Trust programmes in Africa, specifically the Initiative to Develop African Research Leaders (IDeAL) programme, embedded in the KEMRI-Wellcome Trust Research programme.

## 2 Aims and objectives

The overall aim of this initiative is to build research capacity amongst a select cohort of early- and mid-career female researchers from a range of disciplines in South-Central Asia and East Africa through a phased, competitive, training and research fellowship programme. The platform seeks to enhance capacity in bespoke research areas including sexual and reproductive health; pregnancy, perinatal, and newborn care; maternal and child nutrition; child and adolescent growth and development; climate change and its impact on maternal, newborn, and child health; and the implementation and monitoring of HHSDGs.

## 3 Programme development: methodology

The Supporting Women in Science programme was developed to cater to the unique needs of female researchers, bearing in mind the limitations and time constraints of full-time employment. The programme includes an online, distant learning course, which allows participants across multiple time zones to learn at their own pace. To develop such an innovative and inclusive programme, it was crucial to incorporate input from experts, create a comprehensive structure, develop a communications strategy to increase programme reach, monitor progress and sequentially improve capacity.

### 3.1 Development and oversight of advisory committees

To create and manage such an initiative at scale, required input from a broad range of experts to widen the programme's scope and achieve maximum diversity.

An advisory committee, consisting of representatives from the Aga Khan University (AKU), Oxford and key partner institutes, was established to provide guidance, oversight, and strategic direction for the programme. A women-centric steering committee was also established to identify specific challenges and capacity gaps faced by women in research. Their inputs, resources and networks facilitated the development of the programme structure, phases, and course curriculum for trans-disciplinary capacity building.

The all-women steering committee, composed of 12 accomplished women, showcases their extensive expertise in their respective fields. Most members serve as heads of departments in disciplines such as gynaecology, surgery, paediatrics, nursing, and medicine. The committee also includes directors of pioneering initiatives like the Institute for Human Development and the Centre of Excellence in Women and Child Health at the Aga Khan University. They are dedicated to building the capacity of women scientists and, as established leaders in their careers, they are committed to contributing their time to this cause, meeting twice a year to discuss progress.

The steering committee's task is to implement the planning and execution of the strategic plan and ensure the project's objectives are met. It also promotes a culture of continual improvement by implementing necessary adjustments and incorporating feedback into the programme structure. Additionally, the committee's input was crucial in designing the project's phases and engagement strategies. It has sought buy-in from network members, especially regarding access to the core and elective curricula from their respective platforms. The committee made decisions regarding the curriculum, eligibility criteria, research tracks, and programme milestones. Finally, the committee members agreed to participate as mentors and were asked to put forward other researchers within their networks for mentorship.

### 3.2 Programme structure

**Figure 1:**
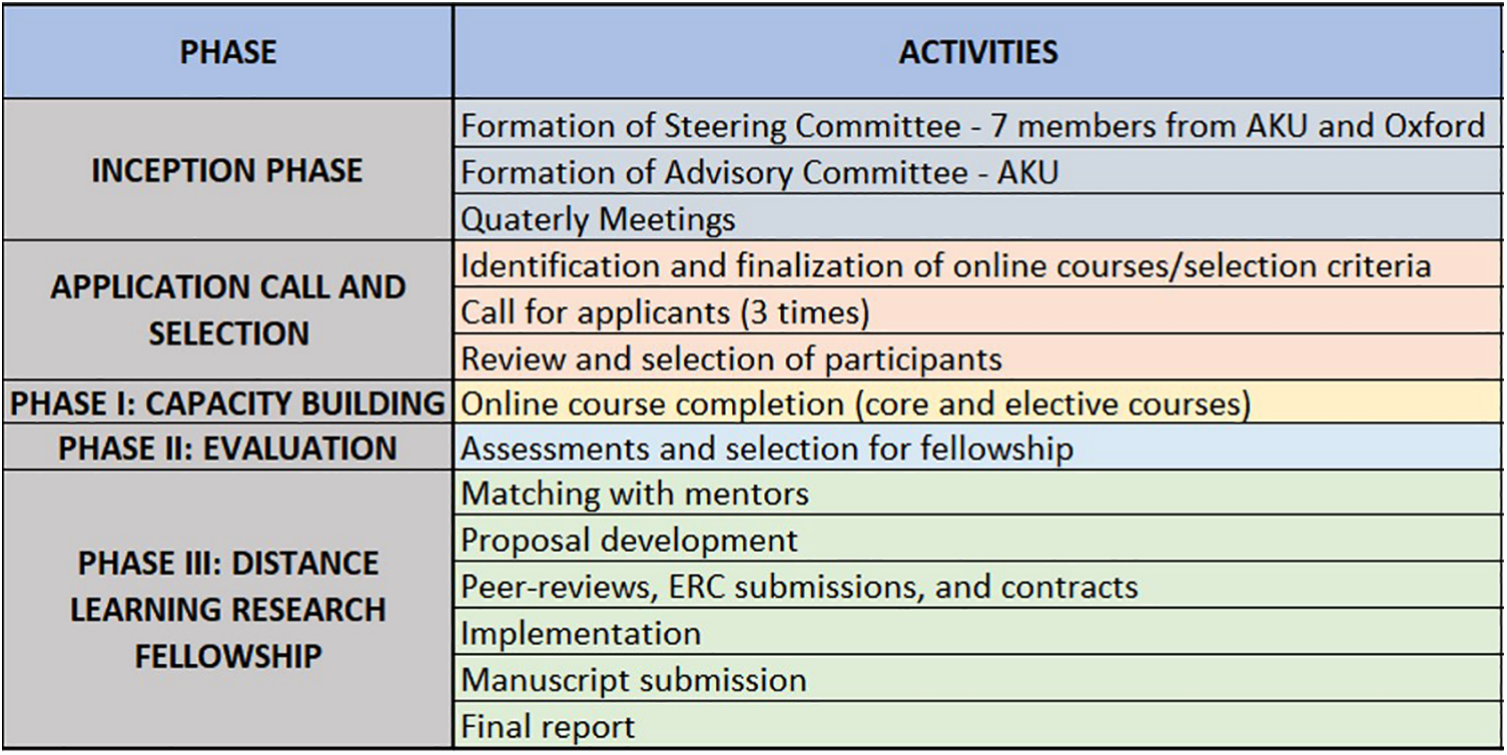
Programme phase activities.

The consultative process with the advisory and steering committees finalised the programme structure as a phased approach to allow for sequential growth and progression of female researchers. Figure 1 illustrates the programme development and phased timelines.

• Phase I: Nomination of early- and mid-career female researchers in a tiered programme of capacity enhancement through online, self-learning modules based on existing high-quality courses.
• Phase II: Evaluation of participants based on a combination of participatory analysis and final summative assessment to serve as a shortlisting criterion for the next phase.
• Phase III: Mentored distant research fellowships under the supervision of AKU and Oxford mentors. Each fellowship entails developing a research proposal and request for financial support up to $20,000.

### 3.3 Programme

#### 3.3.1 Programme announcement and call

A formal call for applications to the “Supporting Women in Science” programme is made on a rolling basis; disseminated through AKU mailing lists, local and international university networks and emails, websites of partner institutions, Higher Education Commission platforms, networks of the steering and advisory committees, and social media. The call is embedded with an information brochure detailing the eligibility criteria and programme timeline, as well as a link to Google forms to facilitate quick and easy responding. Applicants are asked to submit their résumé along with a brief research intent and a letter of recommendation to assess their potential within the scope of the programme. Further dissemination is conducted through regular webinars to create interest in the programme, answer concerns from applicants, and provide information about the application process.

#### 3.3.2 Programme eligibility

The applications received for each call/cohort are assessed based on predefined eligibility criteria, developed by the advisory and steering committees. Eligibility is defined as early- to mid-career women with 0–10 years of experience in working with research and academia. Eligible employment status included junior faculty position, postdoctoral fellows, and research managers in research or academic institutes from South and Central Asia and East Africa with careers in HHSDG-based research. Most participants were expected to have a professional degree or a masters.

#### 3.3.3 Programme orientation

The candidates shortlisted based on the defined eligibility criteria are given a comprehensive guidebook that details the programme structure and requirements. A Zoom call is then organised for each cohort to familiarise the participants with the programme at as convenient a time as possible given the different time zones involved. The participants are provided with specific details about the timelines, core and elective curricula, enrolment in online modules, reporting mechanisms, communication platforms including Slack, and evaluation strategies. The session is interactive to clarify concerns, answer queries, and avoid any misunderstandings.

#### 3.3.4 Phase I: capacity enhancement through online, self-learning courses

The first phase of the programme lasts 6 months and focuses on building academic capacity through self-paced, online courses/modules, which are either “mandatory” or “elective”. The courses are multidisciplinary: obstetrics and gynaecology, perinatal medicine, neonatology, reproductive health, population and public health, nutrition, environmental health and climate change, SDGs, and research methodologies. As such, they move beyond a strict health focus to encompass other areas of health-related SDGs and social sciences to provide a holistic approach towards interdisciplinary research.

The courses were selected based on an identified need for capacity building in bespoke areas, especially research methodologies and analysis. Unlike in high-income countries, students in many LMICs are often introduced to research only towards the end of their undergraduate programs, typically when they are required to complete research projects for graduation (21). Due to the limited career opportunities in research, many students engage in these projects solely to fulfil academic requirements, rather than as a step toward a defined research career and hence often find themselves inadequately prepared and in need of intensive mentoring from senior researchers (21). However, LMIC institutions typically have a limited number of experienced research mentors, creating a gap between theoretical knowledge and practical application—even in cases where research methods are included in the curriculum (22). Thus, the quality of education in research is not optimal and many individuals, though qualified, do not have the required knowledge and skills, highlighting a need to build capacity.

Against this backdrop, the course curriculum, designed by the advisory and steering committees allows participants to acquire not only thematic skills and knowledge but also to participate productively and fully in the analysis and interpretation of data. This involves the statistical skills required to work with larger and more complex databases as well as to interrogate datasets independently. To facilitate course access, we worked with our steering and advisory committees to mobilise their resources to secure free access for our participants. We further collaborated with Coursera to create an AKU-Coursera programme that provides complete access to the selected participants allowing them to do additional courses, which are not part of the programme, at their own discretion.

The core curriculum comprises of six mandatory courses (Table 1):

• INTERGROWTH-21st: Maternal, foetal, and newborn growth monitoring; preterm infant feeding and growth monitoring; and maternal infections offered by the University of Oxford
• Research to publication, offered by the *BMJ*
• Global child health, offered by the University of Toronto
• Introduction to systematic reviews and meta-analysis, offered by Johns Hopkins University through Coursera
• Good clinical practice, offered by National Drug Abuse Treatment Clinical Trials Network
• Sustainable development—interdisciplinary approaches, offered by AKU

**Table 1:** Core curriculum for phase I.

No.	Institution	Course name	Modules	Average completion time	Self-evaluation
1	University of Oxford	INTERGROWTH-21st course on maternal, fetal, and newborn growth monitoring	Assessing newborn size by anthropometry	2 h for each module; 4 h for course completion	Yes
			Assessing maternal anthropometry and weight gain during pregnancy		Yes
		Preterm infant feeding and growth monitoring: implementation of the INTERGROWTH-21st protocol	Background on preterm birth	2 h for each module; 6 h for course completion	Yes
			Gastro-intestinal development in preterm infants		Yes
			Feeding recommendations for the routine care of preterm infants		Yes
		Maternal infections	Overview of maternal infections	2 h for each module; 7 h for course completion	Final quiz for self-evaluation on all modules
			HIV in pregnancy		
			Urinary tract infections in pregnancy		
			Maternal sepsis		
			Syphilis in pregnancy		
			Malaria in pregnancy		
2	BMJ	Research to publication	How to develop and report good research questions	3 h	Yes
			Developing and writing protocols	5 h	Yes
			Choosing the best study design	8.5 h	Yes
			How to do ethical research	8 h	Yes
			How to write a research paper	5 h	Yes
			The essentials of running a clinical trial	12 h	Yes
			Picking the right journal and getting published	6.5 h	Yes
			Avoiding scientific misconduct	6 h	Yes
3	University of Toronto	Global child health	Introduction to global child health	4 h	Yes
			Issues and Interventions in maternal and neonatal health	10 h	Yes
			Concepts of health in the under-five child	10 h	Yes
			Health priorities in the school-age child and adolescent	8 h	Yes
			Early childhood development	4 h	Yes
4	Johns Hopkins University	Introduction to systematic review and meta-analysis	Introduction	1 h	No
			Framing the question	2 h	Yes
			Searching principles and bias assessment	2.5 h	Yes
			Minimizing bias, qualitative synthesis, and interpreting results	2 h	Yes
			Planning meta-analysis and statistical methods	2.5 h	Yes
			Final peer review	1 h	Yes
5	National Drug Abuse Treatment Clinical Trials Network	Good clinical practice	Introduction	0.5 h per module—complete course takes 6 h	No
			Institutional review boards		Yes
			Informed consent		Yes
			Confidentiality and privacy		Yes
			Participant safety and adverse events		Yes
			Quality assurance		Yes
			The research protocol		Yes
			Documentation and record-keeping		Yes
			Research misconduct		Yes
			Roles and responsibilities		Yes
			Recruitment and retention		Yes
			Investigational new drugs		Yes
6	Aga Khan University	Sustainable development—interdisciplinary approaches	Introduction to sustainable development	1 h	Yes
			History and theoretical backdrop	2 h	Yes
			National development plans and possibilities	3 h	Yes
			Introduction to climate change and action	2 h	Yes
			Knowledge generation prospects—learning from the development context	2 h	Yes
			Learning from the community and experts—panel	2 h	Yes

The elective curriculum, which allows participants to enhance capacity in areas of their own interest, comprises courses organised in five domains:

1. Research Methodology/Data Analysis
2. Maternal, Newborn, Neonatal, Women's Health and Nutrition
3. Health in Conflict, Crises, Violence
4. Global Health/Public and Population Health
5. Environmental Health/Climate Change, Health, and Development

Each domain has a broad selection of courses that enable participants to make learning choices based on their research/learning interests. They are required to select any three domains and enrol in one course from each. Table 2 shows the courses provided in each domain.

**Table 2:** Elective course catalogue.

No.	Course name	Institution	Modules	Links
Research methodology/data analysis				
1	Basic statistics	University of Amsterdam	8	https://www.coursera.org/learn/basic-statistics?#about
2	Qualitative data collection methods	Emory University	6	https://www.coursera.org/learn/qualitative-data-collection-methods
3	Qualitative data analysis with MAXQDA software	Emory University	7	https://www.coursera.org/learn/qualitative-data-analysis-with-maxqda-software
4	Summary statistics in public health	Johns Hopkins	6	https://www.coursera.org/learn/summary-statistics
5	Simple regression analysis in public health	Johns Hopkins	5	https://www.coursera.org/learn/simple-regression-analysis-public-health
6	Multiple regression analysis in public health	Johns Hopkins	5	https://www.coursera.org/learn/multiple-regression-analysis-public-health
7	Qualitative research design	Emory University	6	https://www.coursera.org/learn/qualitative-research-design
8	Hypothesis testing in public health	Johns Hopkins	6	https://www.coursera.org/learn/hypothesis-testing-public-health
9	Epidemiology: the basic science of public health	University of North Carolina	6	https://www.coursera.org/learn/epidemiology
10	Study designs in epidemiology	Imperial College London	4	https://www.coursera.org/learn/study-designs-epidemiology
Maternal, newborn, neonatal, women's health and nutrition				
11	Childbirth: a global perspective	Emory University	6	https://www.coursera.org/learn/childbirth
12	Nutrition and lifestyle in pregnancy	Ludwig-Maximilians-Universität München	4	https://www.coursera.org/learn/nutrition-pregnancy
13	Global quality maternal and newborn care	Yale University	8	https://www.coursera.org/learn/global-quality-maternal-and-newborn-care
Health in conflict, crises, violence				
14	Health in complex humanitarian emergencies	Emory University	4	https://www.coursera.org/learn/health-che
15	Non-communicable diseases in humanitarian settings	University of Copenhagen	3	https://www.coursera.org/learn/non-communicable-diseases-in-humanitarian-settings
16	Confronting gender based violence: global lessons for healthcare workers	Johns Hopkins	4	https://www.coursera.org/learn/gender-based-violence
17	Public health in humanitarian crises 2	Johns Hopkins	7	https://www.coursera.org/learn/humanitarian-public-health-2
18	Operational research for humanitarians	University of Geneva	5	https://www.coursera.org/learn/research-humanitarian
Global health/public and population health				
19	Global disease masterclass (specialization)	Imperial College London	12 across 3 courses	https://www.coursera.org/specializations/gmph-global-disease-masterclass?specialization
20	Global health challenges and governance (specialization)	Imperial College London	16 across 3 courses	https://www.coursera.org/specializations/global-health-challenges-governance
21	Essentials of global health	Yale University	10	https://www.coursera.org/learn/essentials-global-health
Environmental health/climate change, health, and development				
22	Environmental health: the foundation of global public health	University of Michigan	4	https://www.coursera.org/learn/environmental-health-the-foundation-of-global-public-health
23	Environmental hazards and global public health	University of Michigan	4	https://www.coursera.org/learn/environmental-hazards-and-global-public-health
24	Human health risks, health equity, and environmental justice	University of Michigan	4	https://www.coursera.org/learn/human-health-risks-health-equity-and-environmental-justice
25	Climate change, sustainability, and global public health	University of Michigan	4	https://www.coursera.org/learn/climate-change-sustainability-and-global-public-health
26	Climate change mitigation in developing countries	University of Cape Town	6	https://www.coursera.org/learn/climate-change-mitigation
27	Act on climate: steps to individual, community, and political action	University of Michigan	7	https://www.coursera.org/learn/act-on-climate

Despite the program being online and asynchronous, a community of practice is created using discussion boards for courses to allow participants to interact and share thoughts and ideas. Further, “Slack”, a communication and collaboration platform, allows participants to organize conversations into channels, making it easier for groups to communicate on specific topics, projects, or teams. These online tools facilitate communication and allow interchange of knowledge and ideas among women scientists from diverse backgrounds.

#### 3.3.5 Phase II: evaluation

Successful candidates from Phase I (defined as those who complete all coursework within 6 months) are evaluated based on a combination of participatory analysis and final summative assessment on the core curriculum. The question bank for the programme, developed in partnership with the SickKids Centre for Global Child Health, Canada, covers all modules of the core courses, and includes multiple choice questions and short answers.

The bank developed was shared with experts in the relevant fields of obstetrics and gynaecology, perinatal medicine, neonatal paediatrics, reproductive health, research methodologies, and public health for content validation and grading. A pilot test was also conducted with a similar target group to assess the suitability of questions and time limits; questions which were deemed unsuitable were discarded.

The Phase II evaluation test is based on 50 questions consisting of 30 multiple choice questions, 10 true and false, and 10 short answers with mixed difficulty levels to be completed in an hour. Scoring is on a 100 grade-point scale with an 80% cut-off to move to the next stage. All participants who pass the exam then undergo an English language proficiency test, which measures their skills and is indicative of future performance in developing and writing proposals, as well as scientific writing.

Phase II is conducted through two digital-based platforms: AKU's Virtual Learning Environment (VLE) and the English Language Enhancement Network (ELE-NET), which is an in-house testing service comparable to other standardised tests such as IELTS and TOEFL. These digital platforms allow administration of a synchronous online test in a proctored environment using dual-device monitoring and a lockdown browser. Test runs are conducted prior to the examination to verify feasibility of the exam modality and to resolve any technical issues to ensure a smooth experience for participants.

A summative evaluation then defines eligibility for “Phase III - Distance Learning Research Fellowship”. The top applicants proceed to the next round for research fellowships mentored by AKU and Oxford faculty.

#### 3.3.6 Phase III: mentored fellowship

Phase III of the programme aims to build the research capacity of those women selected in Phase II through a 18-month mentored fellowship encompassing:

• Pairing with faculty mentors in relevant fields of research
• Cultivating a productive relationship with mentors through regular online meetings and feedback
• Developing a research protocol with supporting documentation (e.g., budget, ethical approvals, etc.) for a 1-year project
• Implementing a 1-year research project with rigorous process and outcome evaluation
• Disseminating research findings and progress through various platforms including conferences, publications, and presentations with mentor support
• Developing leadership and management capacity for professional development

A mentor list, prepared in consultation with the steering committee, includes faculty from AKU, Oxford and other reputed institutes in the following disciplines and beyond: maternal, neonatal, child, and adolescent health; early childhood development; environmental health and climate change; mental health; nutrition; digital health; family planning; sexual and reproductive health; infectious diseases; non-communicable diseases; service delivery; health policy, political economy, and health economy; medical education; and occupational health. Committee members are asked for nominations within their departments to add to the list and all potential mentors are then contacted to confirm their willingness to act as mentors.

Successful candidates from Phase II (Fellows) are required to fill out a form, which captures their areas of research interest and proposed research questions. This information is crucial in matching Fellows with potential mentors based on alignment of research work and interests. Once the faculty member has agreed to act as a mentor, they establish contact with the Fellow to begin assisting in the development of a research protocol, which must be produced within 2 months. The project is devised by the Fellows while the mentors provide valuable advice especially regarding research methodology and project feasibility, keeping in mind the 1-year time limit and the funding envelope.

The protocol must detail the project background and rationale; aims and objectives; research design and methods; proposed activities/interventions; dissemination, outputs, and anticipated impact; project/research timetable; governance and risk management; ethical considerations and barriers to research; and detailed budget with a USD 20,000 limit. The budget covers costs for all project activities including research personnel, operational costs, logistics, equipment, training, and travel expenses. A budget justification is required to assess the cost-effectiveness of the proposed budget and its relevance to the project activities described in the protocol.

The protocols are assessed through peer-review. They are then revised and approved, taking into consideration the budget. Once ethical approval is secured, a contract is then signed by the mentor and Fellow to secure funding. The next stages encompass implementation, evaluation, analysis and writing, and dissemination activities. Overall, Phase III fellowship period encompasses a period of 18 months.

**Figure 2:**
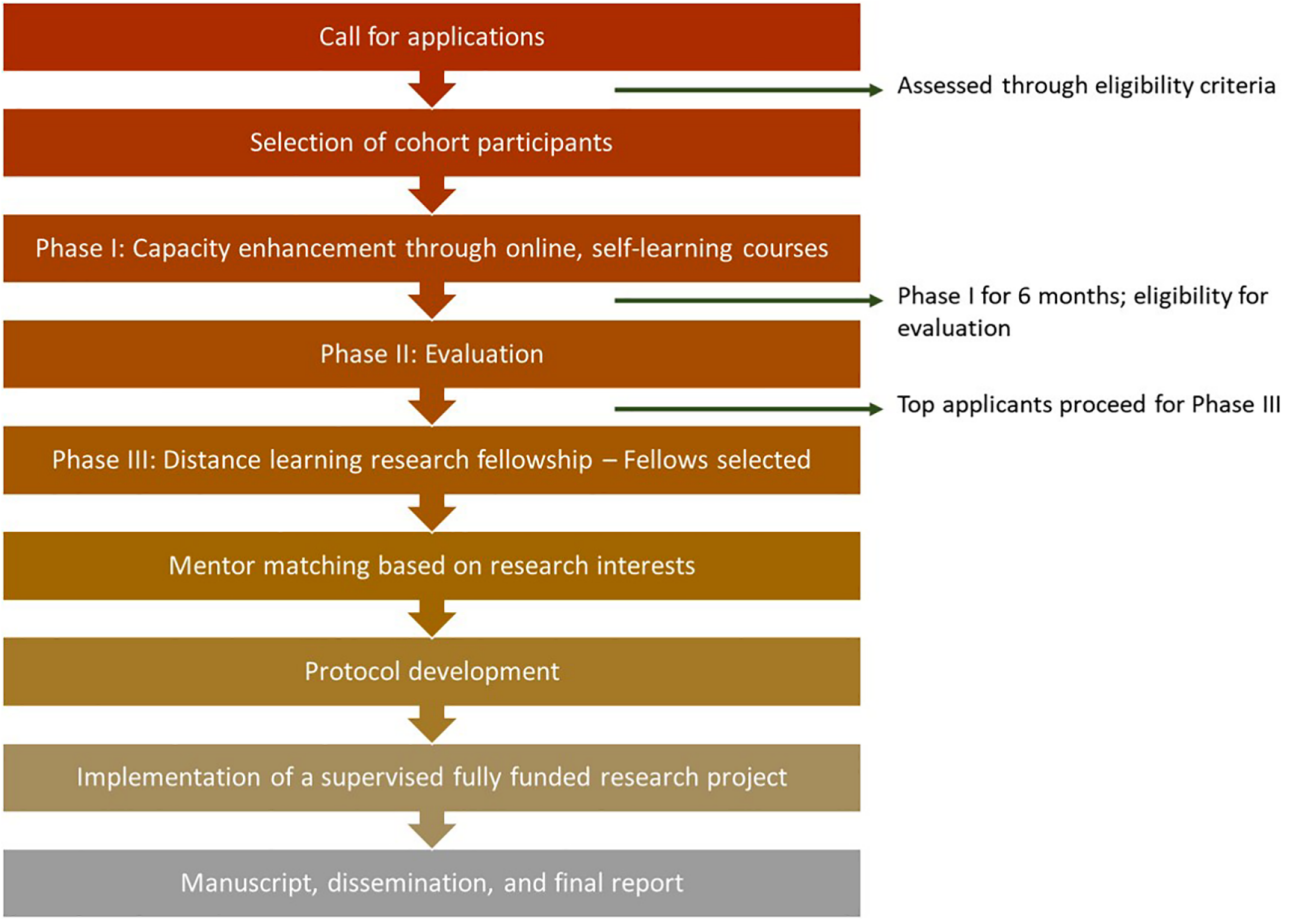
Programme flow.

Within Phase III, the Fellowship Learning Programme also aims to build leadership and management capacity for professional development in selected skills areas such as collaboration; communication; decision-making; project management, and problem solving. Skill levels in these areas are first assessed through the AKU-Coursera platform to calibrate individual, targeted content recommendations so that new skills can be acquired faster. The Fellows share these recommendations with their mentors to help them decide which courses to take. Figure 2 summarises the programme flow.

### 3.4 Reporting and monitoring mechanisms

In Phase I, candidates are required to complete six core and three elective courses within a period of 6 months to proceed to Phase II. This requires comprehensive reporting mechanisms that are easily implemented, maintained, and verified. Once all participants are enrolled in Phase I, they are assigned a unique identifier (WS-ID) for use in official documents and communication. Further, separate Google folders are set up and shared with the respective members to upload and update their required documentation. For monitoring, participants are asked to submit monthly progress reports in their respective Google folders, detailing their activities for the month including courses started, finished, and hours spent. They are also asked to upload any course certificates obtained as proof of completion. As part of our engagement strategy, reminder emails are sent out to promote active engagement. Failure to adhere to the programme requirements or respond to multiple emails leads to removal from the programme. All participants that complete the core and elective course requirements within a period of 6 months, with all necessary reporting including certificate uploads, are progressed to Phase II.

During the fellowship period, to assess compliance and progress, a monthly progress report is shared through individualised Google folders as a live document, which the Fellow is required to update monthly. The format allows the Fellow to report on their meetings with the mentor; the outcomes of each meeting; the stages of protocol development, implementation, analysis, and dissemination; and their progress within the Learning Programme. Similarly, a live expenditure report is required with compulsory quarterly updates to monitor how the funds are being spent. This form of monitoring allows the programme team, mentors, and Fellows to align on progress and provide timely feedback, where necessary.

## 4 Anticipated programme outcomes

The programme phases target capacity building to provide the participants with academic and experiential learning, empowering them to contribute meaningfully to the scientific literature and evidence base. The programme, therefore, enhances their chances of conducting worthwhile and impactful research, interpreting the findings, and publishing for future widespread dissemination. As such, the programme outcomes include:

1. Number of women enrolled in the programme cohort with successful course completions.
2. Number of learning hours and courses completed across programme duration.
3. Number of Fellows inducted with successful completion of their fellowship programme.
4. Number of funded research projects with successful completions.
5. Scholarly output and citation impact generated by participants.
6. Funding and opportunities for research for participants.
7. Frequency of international collaborations and information sharing.
8. Number of Fellows acting as leaders in research based in LMICs (South Central Asia and East Africa primarily, but also including other parts of Africa and South Asia).

## 5 Discussion

The gaps in the research capacity of scientists are profound and require innovative platforms to provide an opportunity for professional advancement. In research and academia, securing grant funding is crucial for career progression as it allows scientists to conduct research, publish relevant papers in (highly cited) journals, and be competitive for further grants and positions (23). Furthermore, grant funding facilitates publication and dissemination of additional research amounting to about one additional article in each of the 3 years following the funding (24). The higher citation metrics and altmetrics by funded researchers suggest that impact goes beyond quantity and that funding fosters dissemination and quality (24). Consequently, if researchers are not successful in acquiring funding, they cannot make a career in science (24).

The SWIS not only provides participants with relevant knowledge and skills through learning but also allows the application of acquired expertise through a grant development, implementation, and evaluation process. This controlled simulation of real-life grant application procedures will enable our female researchers to understand the complexities of such processes and instils confidence in them to apply for their own grants, after completing the programme.

In addition, the programme utilises online, self-paced courses to build capacity. The popularity of online platforms for learning is increasing every year. From 2011 to 2021, the number of learners reached by massive open online courses (MOOCs) increased from 300,000 to 220 million (25). Between 2012 and 2019, the number of hybrid and distance-only students at traditional universities increased by 36%, while the circumstances of the COVID-19 pandemic in 2020 rapidly accelerated that growth by an additional 92% (25). Thus, the wide acceptability of online courses makes our programme widely accessible and convenient for professional women who are already familiar with the process of online, distant learning. Such courses usually require high subscription fees, which women from LMICs cannot afford; hence, the free access to courses through our programme increases its relevance in the LMIC context.

The success of online courses in building professional and academic capacity is widely recorded (26–29). However, building such capacity requires a strong network of mentors who can provide guidance and direction for research inception, implementation, and evaluation. Mentorship can have a profound impact on the success and happiness of a mentee while also providing a sense of fulfilment and enrichment for the mentor (30). The literature supports the value of a mentoring relationship and describes it as an asset to the professional development of an early-career scientist. For example, the productivity, “self-actualisation”, publications, performance, and leadership opportunities for students with mentors may be greater than those without mentors (31). In academic research, mentorship is widely recognized as a means to support the development and retention of faculty who are productive, satisfied, collaborative, and socially responsible. While no randomized trials have assessed the impact of mentorship, systematic reviews suggest that effective mentorship leads to greater faculty productivity (e.g., more grants and publications), faster promotions, and higher retention rates (32, 33).

Given the significant role that mentors play, we employ an exhaustive process to select professionals with a strong research background and expertise in their relevant fields along with mentorship skills. A large qualitative study found that eight themes describe key components of an effective mentoring relationship: (1) open communication and accessibility; (2) goals and challenges; (3) passion and inspiration; (4) caring personal relationship; (5) mutual respect and trust; (6) exchange of knowledge; (7) independence and collaboration; and (8) role modelling (34). Keeping this in mind, we set a strict criterion of selecting mentors for the program to emphasize the importance of strong interpersonal skills alongside research expertise. This will help ensure that mentors are not only knowledgeable but also equipped to provide the personal and professional support needed for successful mentorship.

The mentors are given all the information regarding the programme including the duration of the fellowship, the expectations, and the time commitment required for the role. They are introduced to the potential Fellow along with the details of the Fellow's research interests and research project plans to understand how well their expertise and interests align with the matched candidate. All this information is provided to facilitate an informed decision for mentorship acceptance. Our advisory and steering committees and their networks were crucial in identifying interested professionals who were eager to mentor a cohort of young female researchers. While the mentors guide research direction, they also play a role in the peer-review process. As such, the research proposals developed by each Fellow are assessed through a peer-review process to ensure the quality and feasibility of each project.

We are providing a holistic, research capacity building programme for early- to mid-career female researchers in LMICs. Many studies have established female under-representation in higher education institutions and universities across the globe, and especially in the most powerful or influential posts (15). This plays into societal barriers where ingrained patriarchal attitudes and norms make it difficult for women to tackle male supremacy, disempowerment, and disrespect (35). Patriarchy impacts career progression for women and deprives them of promotions they deserve, solely based on gender discrimination (36). Patriarchal organisations help establish women's low hierarchical positions, giving males more important roles regardless of women's qualifications and education (35). Furthermore, the reigning patriarchal environment does not only impact on female academic output but also on their intellectual and emotional wellbeing (36).

Hence, with such challenges and structural bias, women require capacity building initiatives that can offer them equitable opportunities to progress their career further. However, it is pivotal that such programmes are designed by women who can provide a deeper insight into the challenges faced by their gender in the academic and research world. As such, our all-female steering committee comprised of women in higher positions across partner organisations, utilised their rich experience and contextual knowledge of the challenges and existing gaps to develop this programme. Therefore, Supporting Women in Science is a programme for women, created by women to ensure that it addresses the contextual needs of the target population and allows them to build their capacity. The information helps invaluably to reflect the needs of our learners and deliver a holistic package that sustainably builds capacity for professional and career progression.

Our program has some limitations, as it focuses primarily on building individual capacity rather than organizational capacity, which restricts its ability to address systemic and structural issues that perpetuate gender biases. However, it serves as an important stepping stone by strengthening the skills and capabilities of women scientists, creating opportunities for them to pursue leadership roles and achieve academic success within their institutions. In the long-term, this can potentially position them to effect change from within. Additionally, through our fellowship program, we engage directly with institutions, which allows us to contribute to building some degree of institutional capacity.

In conclusion, our programme provides early- and mid-career female researchers with an excellent opportunity to connect a diverse group of women from Central Asia, South Asia, and East Africa with a quality course in research methodologies and other relevant domains to build their capacity and allow them to link into a wider research network. Additionally, while we aim to build capacity, our Fellowship phase is a wonderful platform to put the research skills acquired to the test and to apply them practically. Hence, a combination of knowledge and practical experience allows candidates to thrive and grow their research capacity to produce exceptional work. Further, unlike a degree or certified course, our program offers the flexibility to adapt to the specific needs of our learners, enabling a more personalized and responsive approach to capacity-building. Moreover, as no similar program has been implemented in this context, we have a unique opportunity to assess its feasibility and apply an iterative process to ensure its success and continuous improvement. In that, we have currently enrolled around 500 women across our cohorts: On average, more than 60% of have successfully completed Phase I. Our later publications will thoroughly evaluate program success and impact and identify lessons learned regarding feasibility, engagement, and utilized strategies and approaches.

Hence, capacity building programmes such as Supporting Women in Science are an essential intervention to support female researchers to gain a much-needed boost to their careers. Programmes tailored to specific needs that target knowledge and skill areas that are lacking develop sustainable capacities for women through affirmative action; provide a step towards equitable gender representation in academia and eliminate power differentials for an inclusive working and research environment. As such, addressing gender inequities in academia not only aligns with the goals of SDG 4 and 5 (quality education and gender equality) but also supports broader efforts towards sustainable development by ensuring that talent and potential are fully realized, regardless of gender.

## Data Availability

The original contributions presented in the study are included in the article/Supplementary Material, further inquiries can be directed to the corresponding author.

## SWIS steering committee

Amina Abubakar, Erum Khan, Fyezah Jehan, Laila Ladak, Lumaan Sheikh, Marleen Temmerman, Pauline Samia, Rozina Karmaliani, Sadaf Khan, Sarah Saleem, Shabina Ariff, and Zainab Samad.
